# Pathology of Insanity

**Published:** 1858-01-01

**Authors:** W. Charles Hood

**Affiliations:** Resident Physician to Bethlehem Hospital


### Art. V.—PATHOLOGY OF INSANITY.

### BASED ON THE POST-MORTEM EXAMINATIONS IN BETHLEHEM HOSPITAL.

BY W. CHARLES HOOD, M.D.,

Resident Physician to Bethlehem Hospital.

(*Continued from* Vol. X. page 384.)

W. C., a male patient, died of paralysis, having been in the hospital two years and two months. On admission he was very thin and in a feeble state, owing, probably, to the small quantity of nourishment he had lately taken. His mental disease had then existed seven weeks, and was evinced by great taciturnity, especially in replying to any questions. When he did speak, it was only to describe various torments that he considered himself undergoing. Want of success in business, and impending poverty, appeared as the exciting cause, influenced by strong hereditary predisposition to insanity. For the first few days after admission he refused any food willingly, and for some time took little more than liquid diet; his obstinacy continued so long, that for upwards of a fortnight it was necessary to give him food by means of the stomach-pump. After this time he sank into a state of complete *melancholia attonita*, rarely speaking, but occasionally giving evidence that he understood what was said to him, and was conscious of passing events; appetite and willingness for food returned, and he would remain for hours sitting up in bed, reading page after page of any book that was given to him, perfectly unconscious of what he was reading. His symptoms varied very little during a protracted illness; weakness gradually increased, paralysis ultimately overtook him, and he died helpless in body and childish in mind.

*Autopsy.*—All the bloodvessels, both of the membranes and brain, extremely turgid. Slight partial opacity of the arachnoid on the convexities of the cerebral hemispheres. Serous infiltration of the pia mater, and much fluid in the basis of the skull, after removal of the brain. Lateral ventricles of the brain distended with clear fluid, estimated at two ounces in each.

The right lung uniformly and firmly adherent, so that it was torn through in drawing out the contents of the thorax; a few very hard small bodies were scattered irregularly through it, and some cavities, not larger than small peas, with loose chalky matter, were discovered, but the lung was otherwise healthy and pervious to air throughout.

The left lung was healthy, and firmly adherent at its posterior aspect.

The pericardium was universally and closely adherent to the heart, the connexion being obviously of ancient date. During two years of this patient's residence in the hospital no peculiarity had been noticed in his pulse, nor any symptoms referable to the heart, which in structure was perfectly healthy.

The liver was soft, so that the substance yielded readily to the pressure of the finger.

The middle portion of the colon (transverse arch), was nearly twice its usual length, bending down after the hepatic flexure to the pubes, and then ascending to the left hypochondrium. (This peculiarity has been observed in a few other instances in the Bethlehem examinations.) The colon was greatly distended with flatus, except in its ascending portion. The body generally well nourished; there was much fat in the omentum and about the abdominal viscera, and a stratum of an inch deep under the integuments of the abdomen.

M. C., a female patient, died of gradual exhaustion, after a residence in the hospital of thirty-one years. Until the year 1854, (twenty-nine years after admission,) this patient was the subject of recurrent mania, having periods of excitement and violence, followed by seasons of depression, the mania quickly succeeding the melancholia, without any lucid interval. During the last two years of her life the maniacal symptoms were constant, and she died, the excitement and irritation peculiar to that stage, yielding only to physical exhaustion.

*Autopsy.*—Great general emaciation. The external vessels of the head empty; the internal turgid. Slight serous infiltration of the pia mater. The lateral ventricles enlarged, and containing each not less than an ounce of fluid. Much fluid about the velum and pineal gland. No morbid change was observed in the substance of the brain.

Both lungs connected to the thoracic parietes by old strong adhesions of considerable extent; they were both somewhat congested and thickened in their posterior aspect, from long continuance in the recumbent position, but not otherwise diseased. The heart was healthy. No other change was observed in the abdominal viscera.

G. G., a female patient, died of phthisis pulmonalis, aged 62, after residing in the hospital thirty-three years. Her occupation had been that of a milliner. Her symptoms were maniacal, and she was under the influence of many delusions, principally of an extravagant character, inducing her to expect court and attention from her fellow-patients and the officials of the hospital. The physical disease which caused her death was severe in its character, but of short duration; the delusions and mental peculiarities remaining unaltered to the last moment of her life.

*Autopsy.*—Skull-cap thin and devoid of blood; considerable quantity of clear fluid escaped on its removal. The substance of the brain soft; an entire absence of bloody points. The lateral ventricles contained about two ounces of serous fluid.

Left lung small, and much compressed by a spinal curvature; the structure of the lung healthy. Apex of the right lung completely altered by the substitution of a tubercular mass for the healthy substance, and throughout the other portion of the lung tubercular deposits were scattered. Heart apparently healthy. No disease observed in the abdominal viscera.

C. H., a male patient, died of general paralysis, aged 27, after thirteen months' residence in the hospital. By occupation, a jockey and horse trainer, he had been exposed to, and indulged in excesses of almost every description; an easy temper and naturally weak intellect offering little opposition to temptation. On admission, evident symptoms existed of approaching general paralysis, although they were not sufficiently stamped to render him ineligible by the rules of the hospital. The disease quickly showed itself, advancing rapidly stage by stage in the following order: Irritability; tremor of the upper lip and edges of the tongue; unsteadiness in gait and articulation; mental excitement and extravagant conversation; delusions connected with personal power and possessions; loss of memory; inattention to habits of cleanliness and self-respect; perfect dementiæ and death.

*Autopsy.*—General emaciation of the frame, with entire absence of fat in the thorax and abdomen. Little blood in either the external or internal vessels of the head. General thickening of the arachnoid, with infiltration of the subjacent tissue over the entire convexity of both cerebral hemispheres. The substance of the pia mater was filled with fluid, like a sponge; this membrane adhered so closely that, in detaching it, portions of the grey substance were torn away in several places. Between two and three ounces of perfectly limpid fluid in the two lateral ventricles. Vascular congestion, with partial solidification to an extent not very considerable, at the posterior aspect of both lungs.

The heart small, not containing much blood. No morbid appearance in the abdomen.

L. D., a female criminal patient, died, aged 66, of exhaustion and the effects of age, after a residence in the hospital of thirty-three years.

This woman was tried at Worcester for the crime of infanticide, and acquitted on the ground of insanity. During her prolonged residence in the hospital she was, with two exceptions, uniformly cheerful, and endeavoured to make herself and those around her comfortable. During these two exceptional periods she suffered from melancholia and attempted suicide, each attack being preceded by febrile excitement. A short time previous to her death she again became melancholic and restless; her mental symptoms appeared not only to be influenced, but much aggravated, by the physical weakness attending age, and in this state she sank and died.

*Autopsy.*—The skull-cap of compact bony substance, heavy and rather thick. The internal vessels both of the brain and membranes congested. A convolution of the posterior lobe of the left hemisphere shrunk so as to leave a vacuity of an inch in length by half an inch wide, occupied by serous infiltration of the pia mater. A similar effect in less degree in two or three other situations. Slight general infiltration of the pia mater. The lateral ventricles distended with limpid fluid, at least half an ounce in each.

Slight adhesion of the left lung, with partial consolidation to a small extent of the inferior lobe at its posterior part. The pleura covering the part thus consolidated covered by an effusion of soft slightly adherent fibrin. Two or three small bits of similar consolidation at the back of the right lung, which was not adherent. In the rest of their substance both lungs were healthy. No morbid appearances observed in the abdomen.

M. A. J., a female patient, died of general anasarca and disease of the heart, after seventeen years' residence. She was married, and the mother of three children. When first admitted she was in good bodily health, but under perpetual morbid fear of being murdered. Very restless at night, and fretful during the day. She ultimately became more maniacal, was subject to sudden outbursts of passion and violence, at the same time occasionally (as if induced by fear) she started off to escape some imaginary persecutors. Her conduct was generally childish, and at all times she showed great disinclination to occupy herself. Her conversation was frequently very obscene, and always devoid of any intelligence. Until two years before her death her general health was good; after that time she suffered much from dyspnœa and all the complications of diseased heart. Her mind became gradually more and more imbecile, and she died in a comatose state, though not much more intellectually lost than she had been for many months.

*Autopsy.*—The body not emaciated; there was a moderate share of fat under the integuments, as well as about the viscera.

All the bloodvessels of the brain and membranes extremely turgid. The two cerebral hemispheres presented a remarkable contrast in external appearance. The convolutions of the left were completely flattened by internal pressure, and the pia mater was throughout of a dull pink colour, from intense vascular congestion. Excepting slight infiltration of the pia mater with a clear fluid of a yellowish tint and fulness of vessels, the corresponding parts of the right side were in a natural state. The cerebral substance on the lower lateral and under part of both posterior lobes was extensively and deeply disorganized, this change nearly reaching to the lateral ventricle. It was softened, partially broken down, and mixed with small portions of coagula, yielding to the slightest pressure. The vessels of the surrounding medullary substance were injected to the utmost, and this substance presented in the posterior lobe a slight brownish-yellow discoloration. The bulk of the right hemisphere had been so increased, that the right ventricle was pushed over to the left of the middle line of the skull.

There were old adhesions of both lungs, but no recent disease.

The heart was large and distended with blood; there was some thickening and induration of the left auriculo-ventriculo valve, but not such as to interfere with the circulation.

The kidneys were slightly granulated, with partial thickening and adhesion of the capsule. No other morbid appearance was noticed in the abdomen.

J. S., a male criminal patient, admitted in the year 1830, having been tried at the Central Criminal Court for discharging a loaded pistol at one of his Majesty's subjects, and then acquitted on the ground of insanity. The first symptom of insanity was noticed during a sea voyage six months previously, when he ordered the captain to turn the ship round or they would be lost, he having seen a man in the sun who had given him the counsel. On his return to England he threatened his uncle's life, while under temporary mental irritation and vexation. He was afterwards taken into custody at the entrance to the House of Lords, where he had waited three days, with a loaded pistol and a knife concealed in his sleeve, for the purpose of assassinating the late Duke of Wellington. For many years before his death he considered himself a prophet, and foretold plagues and earthquakes, as God's revenge for his incarceration. His conduct was generally good, and while he maintained the delusions with great pertinacity, he was never unwilling to listen to the arguments of others. He was very anxious for liberty, and though still maintaining his character as prophet, he nevertheless, on the death of the late Duke, sued for his discharge, considering that his crime was expiated. His mental state altered but little during his sixteen years' confinement, when he died of general dropsy, following disease of the liver.

*Autopsy.*—Great general emaciation; the fat had entirely disappeared from the thoracic and abdominal regions. No morbid appearances were noticed in the contents of the cranium.

Partial firm adhesion of the left lung. Considerable but not general tuberculation of the upper and back part of this lung, with a vomica of moderate size. The right lung was healthy, and not adherent. The heart healthy. Immense enlargement of the liver, which was at the same time very heavy. It descended below the cartilaginous margin of the chest, nearly filling the left hypochondrium, and pushed the diaphragm upwards, encroaching so much on the chest that the lungs did not collapse when the pleuræ were opened. This increase of size was caused by the deposition throughout the organ of adventitious masses, varying in size from that of a small pea to a diameter of three or four inches, generally of circular figure, immediately surrounded and continuous with healthy hepatic structure, without any intervening capsule. They were firm, and of whitish colour, and of nearly homogeneous substance; they were flattened on the surface of the liver, and the smaller resembled in colour and consistence the secondary deposits in the liver in cases of cancer. Some absorbent glands near the pancreas, and in two or three other situations, were discovered similarly enlarged. There were numerous hard flattish tubercles, not large, in the great omentum, and still smaller ones in the peritoneum, at the lower part of the abdomen. The hepatic substance connecting the morbid deposits was healthy, and of dark colour from vascular turgescence. It was considerably less in quantity than the amount of the morbid growths. The gall-bladder was moderately full of healthy bile. The abdomen contained dropsical fluid of strong bilious tinge, and the skin was of the same colour. Several ulcers in the cœcum and neighbouring part of the colon. Incipient granular degeneration of the kidneys, with partial adhesion of the capsules.

(*To be continued.*)

---

